# The European Nucleotide Archive in 2021

**DOI:** 10.1093/nar/gkab1051

**Published:** 2021-11-26

**Authors:** Carla Cummins, Alisha Ahamed, Raheela Aslam, Josephine Burgin, Rajkumar Devraj, Ossama Edbali, Dipayan Gupta, Peter W Harrison, Muhammad Haseeb, Sam Holt, Talal Ibrahim, Eugene Ivanov, Suran Jayathilaka, Vishnukumar Kadhirvelu, Simon Kay, Manish Kumar, Ankur Lathi, Rasko Leinonen, Fabio Madeira, Nandana Madhusoodanan, Milena Mansurova, Colman O’Cathail, Matt Pearce, Stéphane Pesant, Nadim Rahman, Jeena Rajan, Gabriele Rinck, Sandeep Selvakumar, Alexey Sokolov, Swati Suman, Ross Thorne, Prabhat Totoo, Senthilnathan Vijayaraja, Zahra Waheed, Ahmad Zyoud, Rodrigo Lopez, Tony Burdett, Guy Cochrane

**Affiliations:** European Molecular Biology Laboratory, European Bioinformatics Institute, Wellcome Genome Campus, Hinxton, Cambridge CB10 1SD, UK; European Molecular Biology Laboratory, European Bioinformatics Institute, Wellcome Genome Campus, Hinxton, Cambridge CB10 1SD, UK; European Molecular Biology Laboratory, European Bioinformatics Institute, Wellcome Genome Campus, Hinxton, Cambridge CB10 1SD, UK; European Molecular Biology Laboratory, European Bioinformatics Institute, Wellcome Genome Campus, Hinxton, Cambridge CB10 1SD, UK; European Molecular Biology Laboratory, European Bioinformatics Institute, Wellcome Genome Campus, Hinxton, Cambridge CB10 1SD, UK; European Molecular Biology Laboratory, European Bioinformatics Institute, Wellcome Genome Campus, Hinxton, Cambridge CB10 1SD, UK; European Molecular Biology Laboratory, European Bioinformatics Institute, Wellcome Genome Campus, Hinxton, Cambridge CB10 1SD, UK; European Molecular Biology Laboratory, European Bioinformatics Institute, Wellcome Genome Campus, Hinxton, Cambridge CB10 1SD, UK; European Molecular Biology Laboratory, European Bioinformatics Institute, Wellcome Genome Campus, Hinxton, Cambridge CB10 1SD, UK; European Molecular Biology Laboratory, European Bioinformatics Institute, Wellcome Genome Campus, Hinxton, Cambridge CB10 1SD, UK; European Molecular Biology Laboratory, European Bioinformatics Institute, Wellcome Genome Campus, Hinxton, Cambridge CB10 1SD, UK; European Molecular Biology Laboratory, European Bioinformatics Institute, Wellcome Genome Campus, Hinxton, Cambridge CB10 1SD, UK; European Molecular Biology Laboratory, European Bioinformatics Institute, Wellcome Genome Campus, Hinxton, Cambridge CB10 1SD, UK; European Molecular Biology Laboratory, European Bioinformatics Institute, Wellcome Genome Campus, Hinxton, Cambridge CB10 1SD, UK; European Molecular Biology Laboratory, European Bioinformatics Institute, Wellcome Genome Campus, Hinxton, Cambridge CB10 1SD, UK; European Molecular Biology Laboratory, European Bioinformatics Institute, Wellcome Genome Campus, Hinxton, Cambridge CB10 1SD, UK; European Molecular Biology Laboratory, European Bioinformatics Institute, Wellcome Genome Campus, Hinxton, Cambridge CB10 1SD, UK; European Molecular Biology Laboratory, European Bioinformatics Institute, Wellcome Genome Campus, Hinxton, Cambridge CB10 1SD, UK; European Molecular Biology Laboratory, European Bioinformatics Institute, Wellcome Genome Campus, Hinxton, Cambridge CB10 1SD, UK; European Molecular Biology Laboratory, European Bioinformatics Institute, Wellcome Genome Campus, Hinxton, Cambridge CB10 1SD, UK; European Molecular Biology Laboratory, European Bioinformatics Institute, Wellcome Genome Campus, Hinxton, Cambridge CB10 1SD, UK; European Molecular Biology Laboratory, European Bioinformatics Institute, Wellcome Genome Campus, Hinxton, Cambridge CB10 1SD, UK; European Molecular Biology Laboratory, European Bioinformatics Institute, Wellcome Genome Campus, Hinxton, Cambridge CB10 1SD, UK; European Molecular Biology Laboratory, European Bioinformatics Institute, Wellcome Genome Campus, Hinxton, Cambridge CB10 1SD, UK; European Molecular Biology Laboratory, European Bioinformatics Institute, Wellcome Genome Campus, Hinxton, Cambridge CB10 1SD, UK; European Molecular Biology Laboratory, European Bioinformatics Institute, Wellcome Genome Campus, Hinxton, Cambridge CB10 1SD, UK; European Molecular Biology Laboratory, European Bioinformatics Institute, Wellcome Genome Campus, Hinxton, Cambridge CB10 1SD, UK; European Molecular Biology Laboratory, European Bioinformatics Institute, Wellcome Genome Campus, Hinxton, Cambridge CB10 1SD, UK; European Molecular Biology Laboratory, European Bioinformatics Institute, Wellcome Genome Campus, Hinxton, Cambridge CB10 1SD, UK; European Molecular Biology Laboratory, European Bioinformatics Institute, Wellcome Genome Campus, Hinxton, Cambridge CB10 1SD, UK; European Molecular Biology Laboratory, European Bioinformatics Institute, Wellcome Genome Campus, Hinxton, Cambridge CB10 1SD, UK; European Molecular Biology Laboratory, European Bioinformatics Institute, Wellcome Genome Campus, Hinxton, Cambridge CB10 1SD, UK; European Molecular Biology Laboratory, European Bioinformatics Institute, Wellcome Genome Campus, Hinxton, Cambridge CB10 1SD, UK; European Molecular Biology Laboratory, European Bioinformatics Institute, Wellcome Genome Campus, Hinxton, Cambridge CB10 1SD, UK; European Molecular Biology Laboratory, European Bioinformatics Institute, Wellcome Genome Campus, Hinxton, Cambridge CB10 1SD, UK; European Molecular Biology Laboratory, European Bioinformatics Institute, Wellcome Genome Campus, Hinxton, Cambridge CB10 1SD, UK; European Molecular Biology Laboratory, European Bioinformatics Institute, Wellcome Genome Campus, Hinxton, Cambridge CB10 1SD, UK; European Molecular Biology Laboratory, European Bioinformatics Institute, Wellcome Genome Campus, Hinxton, Cambridge CB10 1SD, UK

## Abstract

The European Nucleotide Archive (ENA, https://www.ebi.ac.uk/ena), maintained at the European Molecular Biology Laboratory's European Bioinformatics Institute (EMBL-EBI) provides freely accessible services, both for deposition of, and access to, open nucleotide sequencing data. Open scientific data are of paramount importance to the scientific community and contribute daily to the acceleration of scientific advance. Here, we outline the major updates to ENA’s services and infrastructure that have been delivered over the past year.

## INTRODUCTION

The European Nucleotide Archive (https://www.ebi.ac.uk/ena) has, for almost 40 years, been an important feature in the world of nucleotide sequencing data, playing a key role in storage, contextualisation and discovery of these ever-growing datasets. Supporting users both to deposit and to query and fetch data is our priority, and the tools and services that we continue to develop reflect this. The importance of well-structured open data has been thrown into stark relief by the emergence of the COVID-19 pandemic, and the ENA has adapted to meet the new challenges that come with such data rates and volumes. Simultaneously, the building momentum of many biodiversity projects leads to increasing complexity of taxonomy maintenance and sample classification (e.g. BiCiKL: https://bicikl-project.eu/; Darwin Tree of Life: https://www.darwintreeoflife.org/; Aquatic Symbiosis Genomic Project: https://www.sanger.ac.uk/collaboration/aquatic-symbiosis-genomics-project/; UniEuk: https://unieuk.org/).

The European Nucleotide Archive is part of the International Nucleotide Sequence Database Collaboration (INSDC) (1), which ensures that data is captured and mirrored globally, in collaboration with international partners: the United States National Institutes of Health's National Center for Biotechnology Information (NCBI; GenBank and Sequence Read Archive) and the Japanese National Institute of Genetics’ DNA DataBank of Japan (DDBJ) (2, 3). This active collaboration focuses on development of shared data standards and exchange mechanisms to provide the most rich and accurate network of data to the global community. ENA is an ELIXIR Core Data Resource and Deposition Database (4).

In 2021, we continued to expand, improve and adapt our services in response to the needs of the scientific community. In this article, we discuss updates to our submissions interfaces, overhaul of sequence storage infrastructure to meet rising data volumes, and highlight other notable developments in data submission and access tools.

## CONTENT AND SERVICES

We have continued to operate our range of services to support the submission, archiving, presentation and retrieval of open nucleotide sequencing datasets, including hands-on support from our helpdesk team and comprehensive documentation. Table 1 lists ENA services and their entry points.

**Table 1.** Description of ENA services and their entry points

Services	Service entry points	Purpose of service	Link to service
User support	Support form	Contact and feedback to helpdesk service	https://www.ebi.ac.uk/ena/browser/support
	Support documentation	Comprehensive documentation on submission, retrieval and other FAQs	https://ena-docs.readthedocs.io/en/latest/
Data submission	Submission tools	Submission guide and tools for submission management	https://www.ebi.ac.uk/ena/browser/submit
Data access	ENA browser	Tools to search many data types	https://www.ebi.ac.uk/ena/browser/search

In the past 12 months, we have supported 2772 submitters from over 80 different countries. This spans over 3.5 million individual submissions and 7787 studies, including over 2.5 million samples, 2.1 million runs and 1.2 million assemblies.

**Figure 1.**
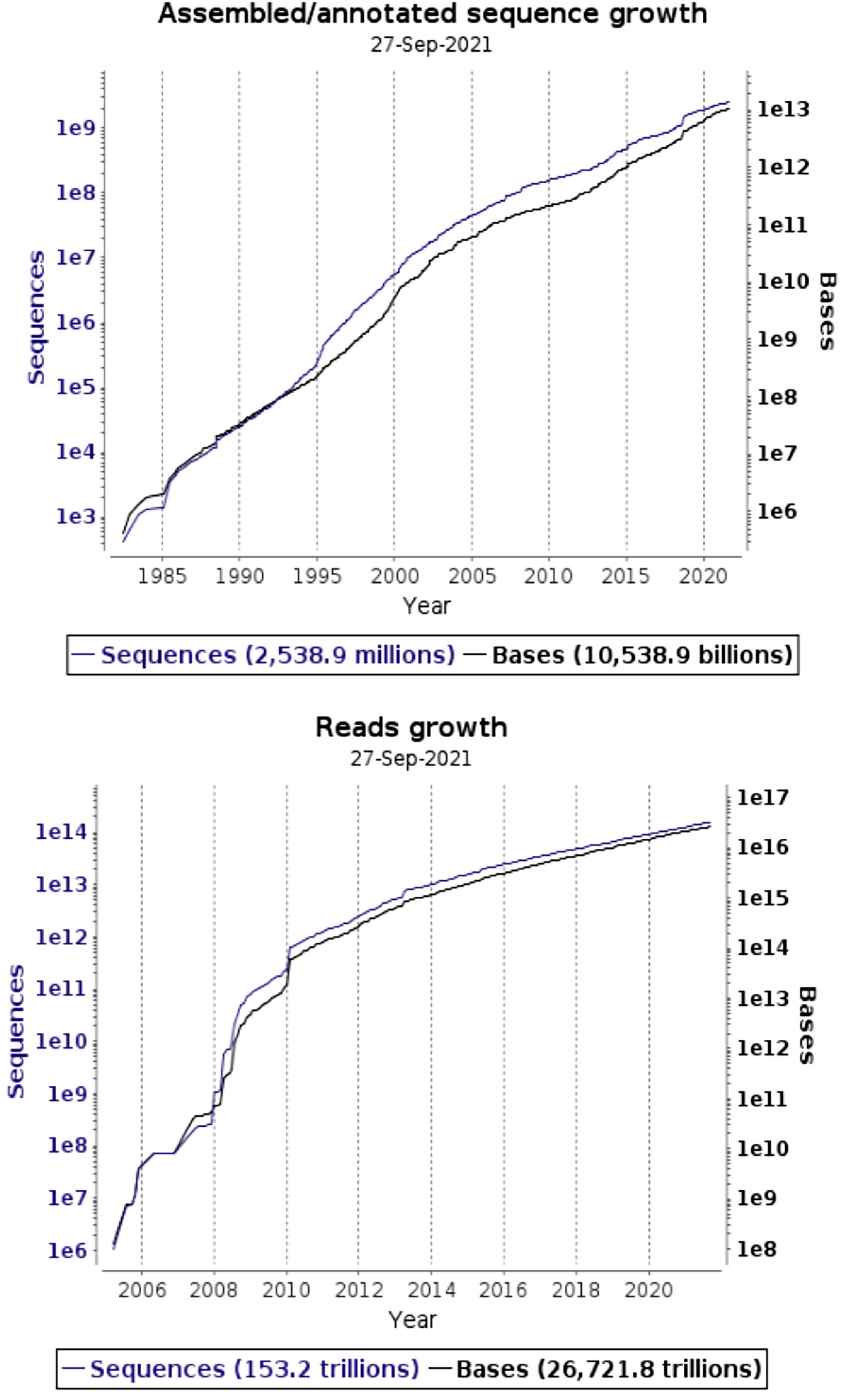
Growth of total data holdings, grouped by assembled/annotated sequences and reads.

At the time of writing, ENA content comprises 2.7 × 10^15^ base pairs of raw sequence data and 2.5 × 10^9^ assembled/annotated sequences (see Figure 1). ENA records offer cross-references to 74 external resources, including newly added links to UniEuk (https://eukmap.unieuk.org/taxonomy) and the Norwegian SARS-CoV-2 national database (https://covid19.sfb.uit.no/) and link to 260 000 publications in the literature.

## EXTENDED AND NEW SERVICES

### Submissions

Our Webin system has provided support for interactive submission of data for some time, providing a user-friendly interface for submission of studies, samples, taxa, raw reads, and assembled and annotated sequences. Within this system, users have access to a rich set of submission templates, which can be downloaded in a simple tab-delimited text format, populated with user data and uploaded to Webin to perform a submission.

**Figure 2.**
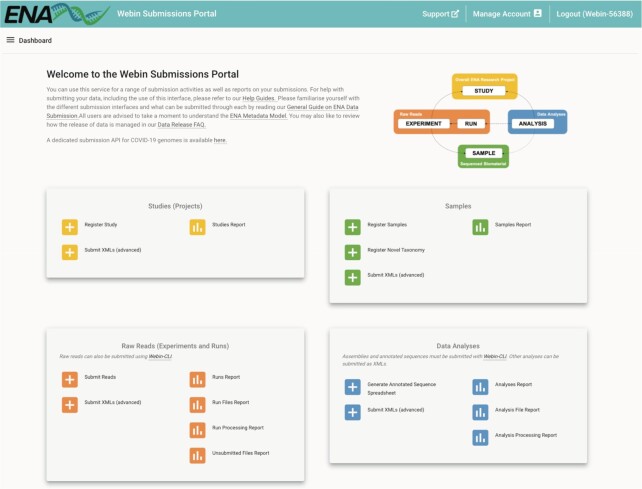
Webin, showing the ‘dashboard’ view.

This year, our Webin system has been overhauled. With a redesigned interface to further improve usability with improved menu layouts (see Figure 2), and added support for new data types and submission workflows in Webin (https://www.ebi.ac.uk/ena/submit/webin), we believe this represents a great step forward. Working in combination with data upload tools such as the Webin File Uploader and users' preferred FTP and Aspera clients, Webin now supports all ENA submission and update workflows. The outgoing service (https://www.ebi.ac.uk/ena/submit/sra/) will be deprecated in late 2021.

We have extended our portfolio of programmatic submissions interfaces for ENA. Building on the flexibility of Webin-CLI, our locally installable tool for validation, management of data file transfers and metadata submission, we have introduced Webin-CLI-REST (https://www.ebi.ac.uk/ena/submit/webin-cli). Removing the need for locally installed tools or file upload steps, this JSON-based RESTful interface provides a simpler, faster, and more reliable validation and submission service. Webin-CLI-REST web endpoints are fully described using Swagger OpenAPI 3.0 (https://www.ebi.ac.uk/ena/submit/webin-cli) and comprehensive documentation and examples can be found at https://ena-covid19-docs.readthedocs.io/en/latest/help_and_guides/webin-cli-rest.html. Currently, this service only supports submissions of SARS-CoV-2 genome assemblies, but we are exploring broadening the scope of the tool to allow data submission from other taxa and of further data types.

In a further offer to those users working with SARS-CoV-2 data, we have released the SARS-CoV-2 Drag and Drop Uploader tool (https://ebi-ait.github.io/sars-cov2-data-upload/). This tool provides an interface for users to drag and drop their data files, accompanied by a metadata spreadsheet, to submit their SARS-CoV-2 raw read data and some non-annotated sequences. Deployed in April 2020, the tool is mainly intended for smaller-scale submissions, reducing the burden on the submitter. Once a user drags and drops their file(s), a User Support Bioinformatician at the ENA is able to provide a direct point of contact for support in the event of validation errors or other issues. Additional tools are offered to support users' SARS-CoV-2 sequence workflows, such as to assist with submissions already made to GISAID (https://github.com/enasequence/ena-content-dataflow/blob/master/scripts/GISAID_to_ENA.README.md) and to prepare bulk submissions (https://github.com/enasequence/ena-bulk-webincli).

**Figure 3.**
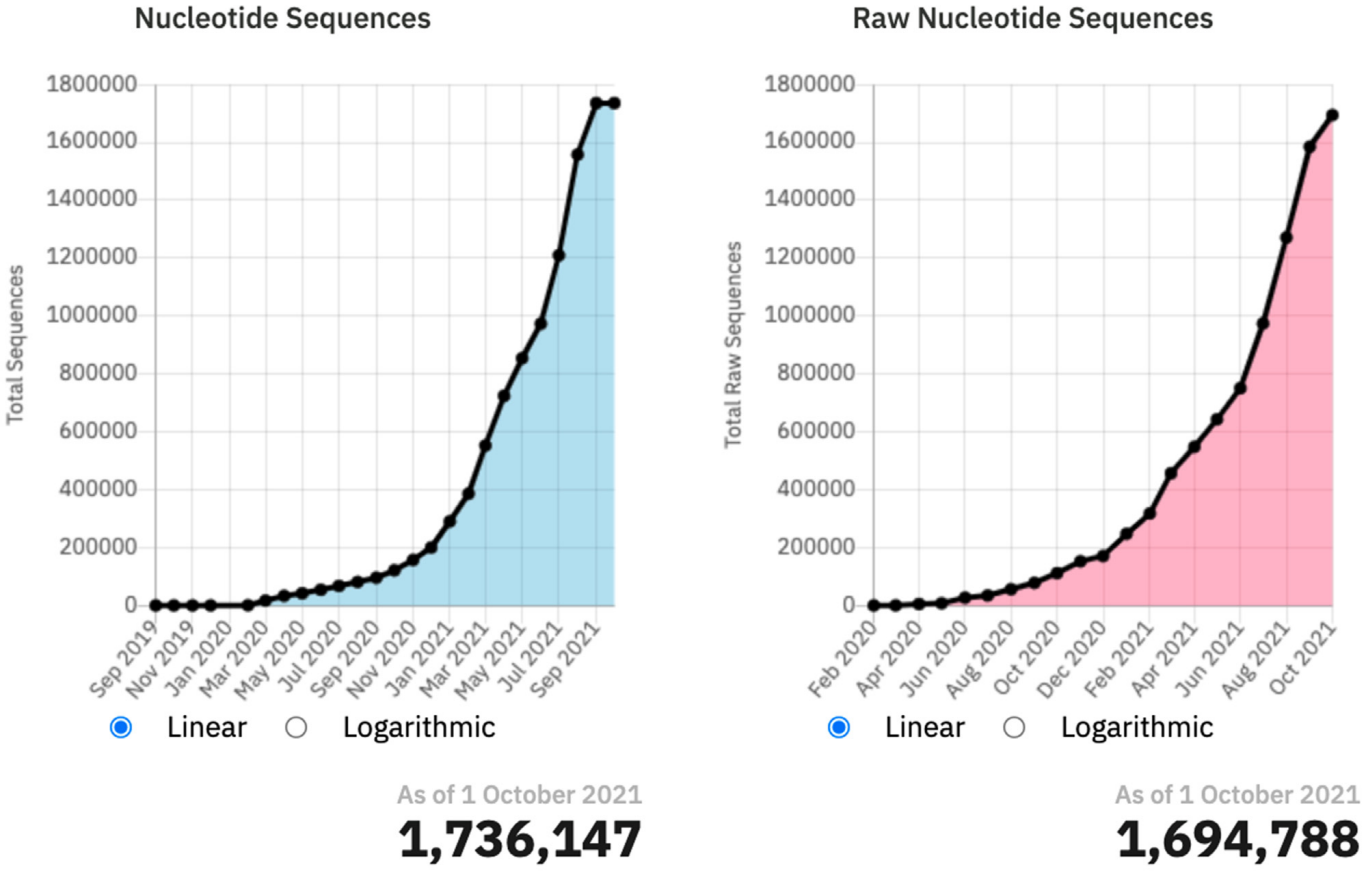
Statistics on data holdings in COVID-19 Data Portal (https://www.covid19dataportal.org/statistics).

Together, these tools have supported substantial efforts to mobilise SARS-CoV-2 data during COVID-19 as part of our European COVID-19 Data Platform initiative (https://www.covid19dataportal.org/the-european-covid-19-data-platform). As of late September 2021, there are 1.66 million SARS-CoV-2 sequences and 1.55 million raw read datasets, as shown in Figure 3. Documentation regarding SARS-CoV-2 submissions has been updated (https://ena-browser-docs.readthedocs.io/en/latest/help_and_guides/sars-cov-2-submissions.html) and support is available via virus-dataflow@ebi.ac.uk.

### Infrastructure

With the exponential growth of sequence records we have sought to alleviate the existing load on internal relational databases and prepare for further scaling. This work, known internally as ‘Direct2FTP’, involves workflows that create a normalised data structure from incoming NCBI and DDBJ that holds, for each contig set, (a) a master record (which is still loaded into the relational database) and (b) a FASTA data file (which is loaded into our object store alongside other data files). This system is currently in place for mirrored data only. Data distribution systems have been updated to be able to handle these new structures and changes are transparent to the end user. However, with this new infrastructure, users will experience more rapid and reliable synchronisation of ENA with the comprehensive global data set.

### Data presentation

Following the retirement of the quarterly ENA sequence release process in March 2020, we have provided further tools to support users wishing to synchronise local data sets with ENA. First, we have launched a system to provide snapshots of the assembled/annotated sequence, coding sequence and non-coding sequence sets. These snapshots provide an index of the records in the data files, including accession, data class, taxonomic division, and last updated date as well as the complete sequences in FASTA format. These snapshots are available on our public FTP site at http://ftp.ebi.ac.uk/pub/databases/ena/sequence/snapshot_latest. Second, we have provided a utility that can be used to compare the full set of public sequences available from ENA against the full set that would have been available on a previous date. This allows users to track which records were added/updated since their last import and which records were deleted (or suppressed). This tool, the ‘snapshot change lister’, is available at https://github.com/enasequence/enaBrowserTools.

Additionally, we have launched the ENA File Downloader (https://github.com/enasequence/ena-ftp-downloader/tree/master/command-line-downloader). This standalone utility allows a user to make systematic calls to ENA’s APIs to poll, retrieve and synchronise content of interest to local storage without requiring any scripting or direct interaction with the APIs. Complementing existing tools such as the ENA Browser, the Python-based enaBrowserTools and the GUI-based ENA FTP Downloader, this new tool adds a further method for downloading and synchronising data and metadata from ENA. Its support for both FTP and Aspera protocols provides flexible data transfer options.

We continue to see high usage of our three-tiered metagenome assembly data structure since its introduction in 2018 (documented here: https://ena-docs.readthedocs.io/en/latest/submit/assembly/metagenome.html). Organising metagenome assemblies into ‘primary metagenome’ (currently 73 060 data sets), ‘binned metagenome’ (22 497) and ‘Metagenome-Assembled Genome (MAG)’ (37 046), we see good compliance with the appropriate data standards (80% of MAG data sets comply with MIMAGS (5) or MIUVIGS (6)). We have improved the discoverability of these data; assemblies submitted to ENA can be retrieved by their ‘assembly type’ and further filtered using associated sample metadata. For example, when retrieving a set of all binned metagenomes with a particular threshold for completeness and contamination, this can be effected in a single API call: = analysis&query = assembly_type = %22binned%20metagenome%22%20AND%20completeness_score%3E90%20AND%20contamination_score%3C5. Data structures for ‘primary metagenomes’ and ‘binned metagenomes’ are not yet exchanged with INSDC, however MAGs are exchanged and available via all INSDC partners. INSDC uses the ‘metagenome source’ annotation to classify assemblies from a metagenomic source and this annotation can be included in searches to include INSDC partner submitted MAGs. For example, a user can fetch all WGS set MAGs submitted across INSDC with the following API query: = wgs_set&query = (assembly_type = %22Metagenome-Assembled%20Genome%20\(MAG\)%22%20OR%20metagenome_source = %22*%22)

## ACCESS TO ENA SERVICES

The entrypoint for access to all ENA services is our homepage (https://www.ebi.ac.uk/ena). Discovery and retrieval of data held in ENA is open to everybody, using the array of tools described in this article and in our documentation (https://ena-docs.readthedocs.io/en/latest/retrieval/general-guide.html). Data deposition requires a Webin account. New Webin accounts can be registered here: https://www.ebi.ac.uk/ena/submit/webin/accountInfo. Further information on data submission can be found here: https://ena-docs.readthedocs.io/en/latest/submit/general-guide.html

## DATA AVAILABILITY

ENA services are freely available at (https://www.ebi.ac.uk/ena). Content is distributed under the EMBL-EBI Terms of Use available at (https://www.ebi.ac.uk/about/terms-of-use).
